# “Dot COM”, a Nuclear Transit Center for the Primary piRNA Pathway in *Drosophila*

**DOI:** 10.1371/journal.pone.0072752

**Published:** 2013-09-09

**Authors:** Cynthia Dennis, Vanessa Zanni, Emilie Brasset, Angeline Eymery, Liang Zhang, Rana Mteirek, Silke Jensen, Yikang S. Rong, Chantal Vaury

**Affiliations:** 1 Clermont Université, Université d'Auvergne, Clermont-Ferrand, France, Inserm, U 1103, Clermont-Ferrand, France, CNRS, UMR 6293, Clermont-Ferrand, France; 2 LBMB, National Cancer Institute, National Institutes of Health, Bethesda, Maryland, United States of America; 3 UMR 1318, INRA-AgroParisTech, Versailles, France; Université Paris-Diderot, France

## Abstract

The piRNA pathway protects genomes by silencing mobile elements. Despite advances in understanding the processing events that generate piRNAs for silencing, little is known about how primary transcripts are transported from their genomic clusters to their processing centers. Using a model of the *Drosophila COM/flamenco* locus in ovarian somatic cells, we identified a prominent nuclear structure called Dot COM, which is enriched in long transcripts from piRNA clusters but located far from their transcription sites. Remarkably, transcripts from multiple clusters accumulate at Dot COM, which is often juxtaposed with Yb-bodies, the cytoplasmic processing centers for cluster transcripts. Genetic evidence suggests that the accumulation of precursor transcripts at Dot COM represents one of the most upstream events in the piRNA pathway. Our results provide new insights into the initial steps of the piRNA pathway, and open up a new research area important for a complete understanding of this conserved pathway.

## Introduction

Transposable elements (TE) are targeted for transcriptional silencing through a mechanism mediated by small RNAs. In animal germ lines, the piRNA (PIWI-interacting RNAs) pathway has been identified as the major mechanism for mounting an effective defense against TE [1], [2], [3]. In *Drosophila*, both germ cells and their associated somatic cells possess a functional piRNA pathway, however the biogenesis of piRNA molecules differs. In both types of cells, a pool of primary piRNAs is presumably processed from long single stranded transcripts. These long transcripts are produced from discrete genomic loci (piRNA clusters) that mainly reside in pericentric heterochromatin enriched in TE or their relics. How precursor transcripts are delivered to processing centers is not understood.

In *Drosophila*, following primary piRNA biogenesis in the germ line, a target-dependent amplification loop called the “ping-pong cycle” produces RNAs effective against TE [2]. A simplified pathway lacking the ping-pong cycle is active in the somatic follicle cells [4], [5], [6], and current models postulate that primary transcripts from piRNA clusters are directed to cytoplasmic Yb-bodies, where primary piRNA biogenesis is thought to take place [7], [8], [9]. The RNA helicase Armitage (Armi) and Tudor domain-containing Yb proteins are the major components of the Yb-bodies, and have been implicated in piRNA biogenesis. In addition, the Zucchini (Zuc) nuclease is necessary for piRNA processing [10], [11]. Finally, mature piRNAs associated with PIWI are delivered to the nucleus for target mRNA cleavage [12], [13]. Consistently, loss of Armi, Yb, Zuc or PIWI function results in de-silencing of transposons in somatic cells [8], [14], [15].

Whereas significant advances have been made in characterizing the downstream events in the piRNA pathway, one of the critical upstream processing events, namely the transport of the primary transcripts to the cytoplasmic processing center, remains largely unexplored. Although the primary transcripts can be detected by RT-PCR [2], [8], direct visualization of the transcripts *in situ* has not been achieved. In this study, we used RNA FISH in combination with immunolocalization to visualize precursor transcripts. We discovered a nuclear structure enriched with cluster transcripts and juxtaposed with cytoplasmic processing centers that we name “Dot COM”. Genetic evidence indicates that Dot COM formation is the most upstream event in the piRNA pathway, following the generation of primary transcripts.

## Results

### Transcripts from the *COM/flam* piRNA locus accumulate in a single nuclear focus, the “Dot COM”

**Figure 1:**
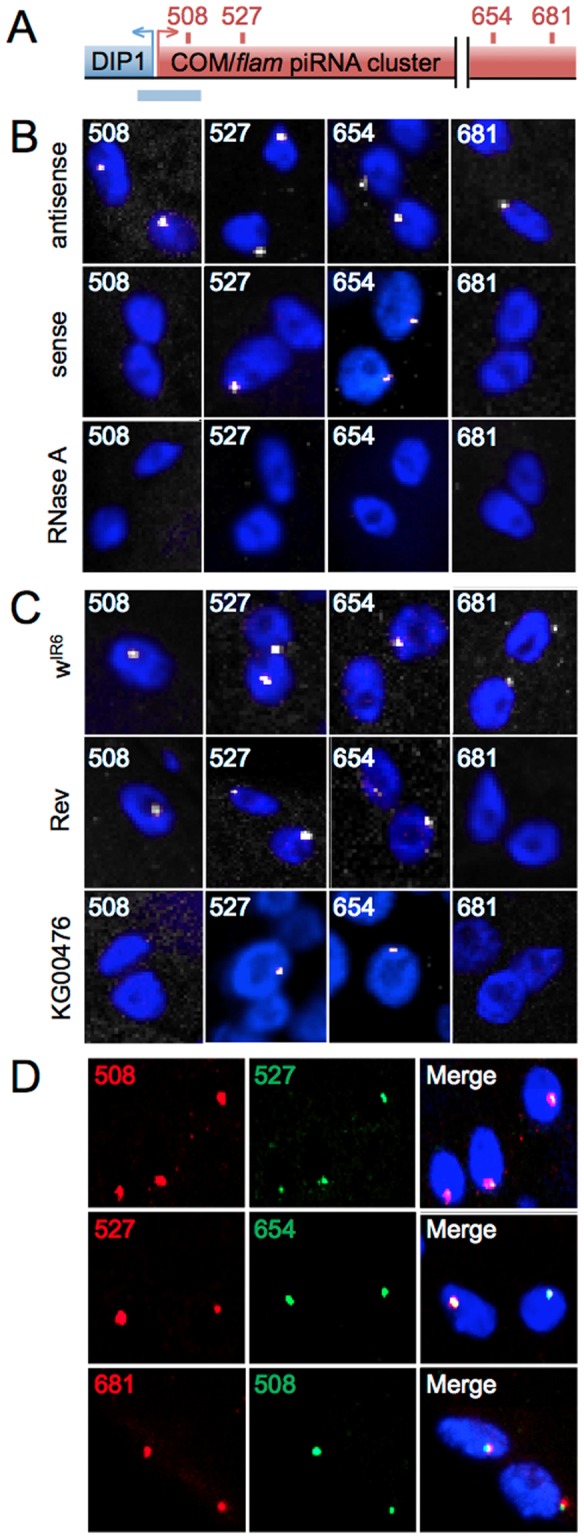
A single focus of *COM/flam* transcripts in follicle cells. (**A**) Genomic structure of the *COM* locus (in red, not to scale) localized at the border of the heterochromatic region of X chromosome, next to the *DIP1* gene (in blue). RNA and DNA probes generated are indicated by red and blue rectangles respectively above and underneath the *COM* locus. (**B** and **C**) FISH staining in ovarian follicle cells with riboprobes 508, 527, 654 and 681 (white) in *ISO1A* line (**B**) and *w^IR6^*, *Rev* and *KG00476* lines (**C**). (**D**) Double FISH staining in *ISO1A* follicle cells with different combinations of the riboprobes indicated. DNA is labeled with Hoechst (blue).

The best-characterized piRNA cluster in Drosophila is located at the *COM/flamenco* (*COM*) locus. This locus displays more than 94% transposon sequences in a non-random orientation, 81, 7% being inserted in minus orientation (proximal to distal orientation). The current model postulates that long unidirectional precursor transcripts traverse the locus and are processed in piRNAs with a marked strand asymmetry for the minus strand that correlates with the strong biased orientation of the transposons (Fig. S1A). To investigate the biological steps between precursor transcript generation and processing of these transcripts into piRNAs, we directly visualized the primary transcripts using FISH. We generated riboprobes, with sizes ranging from 727 to 989 bp that cover four separate regions of the 180 kb *COM* locus (Fig. 1A, and Fig. S1A). The 508 and 681 outer probes are unique to the *COM* locus, whereas the 527 and 654 inner probes share partial homology to other heterochromatic regions in the genome (Fig. S2).

**Figure 2:**
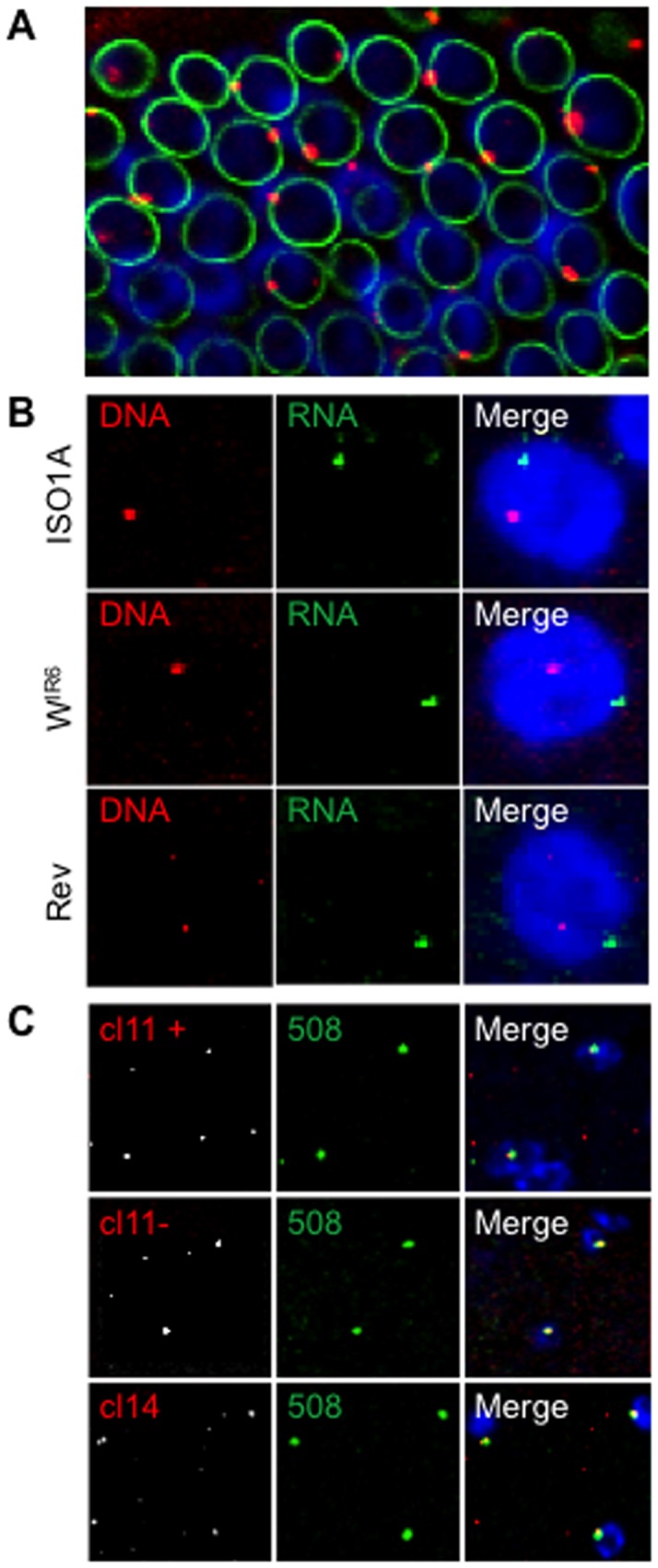
Content and nuclear localization of Dot COM. (**A**) Immuno-RNA FISH staining with antisense 508 riboprobe (red) and anti-lamin antibody (green) in follicle cells of *ISO1A* line. (**B**) DNA/RNA FISH staining for *COM* transcripts (green), the *COM* locus (red) and DNA (blue) in follicle cells in *ISO1A* (top), *w^IR6^* (middle) and *Rev* (bottom) lines. (**C**) Double FISH staining in *ISO1A* follicle cells with different combinations of the riboprobes indicated. cl11 and cl14 probes recognize respectively cluster 11 (both strands indicated by + and −) and cluster 14 [5]. In all rows, the merge RGB image is shown alongside with the individual channels in white and green. DNA is indicated in blue.

Strikingly, signals from the antisense probes at all four regions, which detect the sense transcripts from *COM*, form a single focus in ovarian follicle cells of the *ISO1A* wild type line (Fig. 1B, top line). These signals are exclusively nuclear, as illustrated by immuno-FISH experiments in which lamin detection demarcated the nuclear periphery (Fig. 2A and Video S1). These FISH signals are sensitive to a pre-hybridization RNase A treatment (Fig. 1B, bottom line), supporting the conclusion that the signal represents transcript detection. We name these foci “Dot COM”s. Interestingly, although sense probes of 508 or 681 failed to generate a signal, those from 527 and 654 again produced a visible focus (Fig. 1B, middle line). Since the *COM* locus produces mostly sense transcripts [2], [5], we believe that the signals from 527 and 654 sense probes originated from other piRNA clusters that share sequence homology within the two probe regions (Fig. S2). Our explanation is further supported by additional controls and experiments described below and in later sections.

To further characterize Dot COMs, we repeated the RNA FISH experiments on two *Drosophila* lines in which the *COM*-mediated silencing of the *ZAM* and *Idefix* elements is disrupted (Fig. S1B). We included in the analyses the *w^IR6^* line as another wild type control. Similar to the *ISO1A* wild type line, Dot COM is present in *w^IR6^* (Fig. 1C). In the *KG00476* line, transcription of the *COM* locus is disrupted due to the insertion of a P element upstream of the cluster [16] (Fig. S1B). Consistently, probes from the 508 or 681 regions, which are unique to the *COM* locus, failed to reveal Dot COM (Fig. 1C). In contrast, the Dot COM revealed by probes 527 and 654 is still present, consistent with our previous hypothesis that these signals originate from other homologous regions. The *Rev* line was generated from the *w^IR6^* line as a derivative that is no longer able to silence *ZAM* and *Idefix* [17], [18]. Molecular characterization revealed that *Rev* harbors a chromosomal deletion that eliminates a centromere-proximal region including regions 654 and 681 (Fig. S1B and Zanni *et al*. in preparation). Consistently, only the Dot COM corresponding to the 681 region was absent in the FISH experiments (Fig. 1C), whereas transcripts from the 508 region, which is present in *Rev*, is able to form a Dot COM. In these experiments, a minimum of 100 nuclei was scored for each genotype.

### Dot COM contains COM precursor transcripts located distal to its genomic locus

We consider the transcripts detected by the four antisense probes to be the precursor transcripts from *COM*, which are many kilobases long. Since the four probes are from regions far apart, the prediction is that the Dot COM detected by different probes is actually a single entity. To test this prediction, we repeated RNA FISH using a mixture of antisense probes targeting two different regions, i.e. 508+527, 527+654, and 508+681. As shown in Fig. 1D, in all the combinations, signals from the two regions co-localize very well. We found: 508+527: 97% co-localization (n = 91), 527+654: 91% co-localization (n = 105), 681+508: 95% co-localization (n = 160). This is quite remarkable considering that the 508 and 681 regions are at the ends of the *COM* locus and almost 200 kb apart. These results indicate that most if not all of the transcripts from the *COM* locus, either comprising a single 180 kb transcript or several few kb long, accumulate at the single nuclear focus of the Dot COM.

A logical assumption for the nuclear position of Dot COM is the site of transcription, i.e. the genomic *COM* locus. We investigated this hypothesis by performing a DNA/RNA FISH experiment in which hybridization of a *COM* antisense probe was followed by hybridization of a DNA probe made from the *COM*-adjacent region of *DIP1* (Fig. 1A and Fig. S1A). The DNA and RNA signals did not overlap in any of the 171 nuclei examined (Fig. 2B), indicating that the *COM* transcripts have been actively removed from their site of transcription and accumulate at Dot COM.

### Dot COM contains transcripts from other piRNA clusters

There are many piRNA clusters in the *Drosophila* genome. Therefore, we considered the interesting possibility that transcripts from multiple clusters congregate at Dot COM. As reported above, the riboprobes 527 and 654 recognize repeated sequences found in both *COM* and other heterochromatic regions. These heterochromatic repeats were found mostly around centromeric and telomeric regions. When examined in detail (Fig. S2) they were found to match the genome at minor or major piRNA clusters expressed in somatic follicle cells. [5], [19]. It is therefore striking that a single RNA spot per somatic cell was always revealed by either 527 or 654 probe (Fig. 1B), and suggests that transcripts originating from repeated sequences present within diverse piRNA clusters gather in a common Dot COM.

To further support this hypothesis, we repeated RNA FISH experiments with another set of riboprobes able to uniquely recognize RNAs synthesized from other master piRNA loci known to be active in the follicular cells, cl11 for cluster 11 and cl14 for cluster 14 [5]. Remarkably, both cl11 and cl14 probes identified a single focus in follicle cells (Fig. 2C). Even more interestingly, cl11 signals co-localized with 508 signals from COM in 89% of the nuclei (n = 30) and the degree of co-localization between cl14 and 508 is 90% (n = 50). These results strongly support a model in which Dot COM contains transcripts produced from several piRNA master loci in ovarian somatic cells. In contrast, we observed that transcripts from the telomeric HeT-A elements, which are not regulated by the piRNA pathway in follicle cells [5], did not form nuclear foci (Fig. S3).

### Nuclear Dot COM is often juxtaposed with the cytoplasmic Yb-body

**Figure 3:**
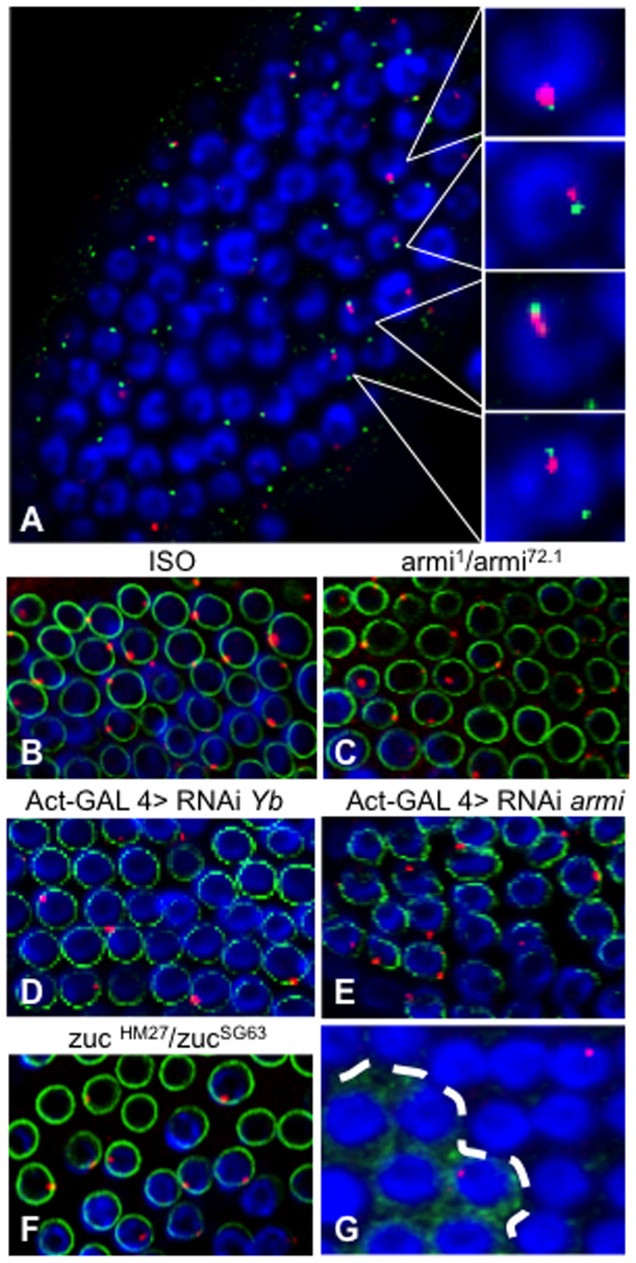
Dot COMs are juxtaposed with Yb-bodies. (**A**) Immuno-RNA FISH staining with antisense 508 riboprobe (red) and anti-Armi antibody (green) in follicular cells. Right panels correspond to higher magnification views. (**B**–**F**) Immuno-RNA FISH staining with antisense 508 riboprobe (red) and anti-lamin antibody (green) in follicle cells of *ISO1A* line (**B**), transheterozygous for *armi* (**C**) and *zuc* (**F**) mutations, and RNAi knockdown of *Yb* (**D**) and *armi* (**E**). (**G**) Immuno-RNA FISH staining with antisense 508 riboprobe (red) and anti-GFP antibody (green) in *piwi* mosaic ovary. Follicle cells carrying two copies of the wild type allele of *piwi* express the GFP marker (green) whereas homozygous *piwi^2^* mutants do not. Dash line separates mutant follicle cells from WT. DNA is stained in blue.

We further characterized the nuclear localization of Dot COM focusing on its relationship with subcellular structures. Using lamin to demarcate the nuclear periphery in FISH experiments with *COM* specific probes, we conclude that Dot COMs reside in close proximity to the nuclear membrane in at least 92% of the nuclei (n = 589) (Fig. 2A and Video S1). From this nuclear periphery, Dot COM precursor transcripts are presumably exported to the cytoplasm for processing into piRNAs. Cytoplasmic Yb-bodies have been proposed to be major processing centers for precursor piRNA transcripts [8], [9], [15]. There are 1–2 Yb-bodies per follicle cell. They often localize next to spherical structures enriched in RNAs, which suggests the involvement of Yb-bodies in RNA metabolism, and are often found close to the nuclear periphery. These interesting features of the Yb-bodies suggest that they may be the structures that receive transcripts from Dot COM. Our hypothesis is strengthened by results from FISH experiments shown in Fig. 3A, in which 69% of Dot COMs (n = 599) were found juxtaposed with an Armi focus, a major component of the Yb-body [8]. Therefore, Dot COM is an RNA-enriched nuclear structure that frequently resides in close proximity to the Yb-body, the presumed processing center for its RNA content. In contrast, Dot COM did not co-localize with other nuclear structures, including the nucleolus, cajal bodies and histone bodies (Fig. S4).

### Dot COM formation is independent of processing and nuclear import of mature piRNAs

Our results have thus far established a new step in the primary piRNA pathway, namely the channeling of the cluster transcripts to Dot COM. We set out to define its genetic relationship to previously characterized steps in the pathway.

Precursor transcripts are presumably transported to the cytoplasmic Yb-bodies, which is consistent with strong co-localization of *COM/flam* transcripts with Armi-marked Yb-bodies. We disrupted the production of Yb-body components using mutations (*armi*) and RNAi (*Yb* and *armi*), however we observed a normal distribution and morphology of Dot COM (Compare Fig. 3B with Fig. 3C–E), even when Yb-bodies are disrupted (Fig. 3D and Fig. S5). We also tested *zuc*-mutant ovaries, as the Zuc endonuclease is thought to be an important player in piRNA processing [10], [11]. In *zuc*-mutant ovaries, however, normal Dot COM is again observed in follicle cells (Fig. 3F). Taken together, these two results suggest that Dot COM formation is independent of cytoplasmic processing of precursor transcripts.

Once processed, piRNAs are loaded onto Piwi and related proteins for their import into the nucleus. Interestingly, in mosaic ovaries containing wild type cells (marked with GFP) and cells homozygous for the *piwi^2^* null allele (lack of GFP), Dot COM can be detected in both types of cells (Fig. 3G), suggesting that Piwi is not needed for Dot COM formation. In summary, the accumulation of precursor transcripts at Dot COM represents the most upstream event in the primary piRNA pathway, barring only the generation of the primary transcripts themselves.

## Discussion

Here we propose that the nuclear structure Dot COM is important for the primary piRNA pathway in somatic follicle cells surrounding the germline. The evidence is at least two fold: (1) Dot COM is a singular structure enriched with transcripts from multiple piRNA clusters, and (2) Dot COM is often juxtaposed with cytoplasmic Yb-bodies, the piRNA processing center. We envision that primary transcripts produced by the clusters might share common features allowing their recognition by cellular machinery, ultimately resulting in their concentration at the nuclear peripheral Dot COM.

For the germ line, it was recently reported that UAP56, a protein previously implicated in mRNA splicing and export, mediates the transfer of cluster transcripts to the peri-nuclear processing machinery [20]. There are several important differences between the UAP56-mediated germline event and the Dot COM-mediated event in the soma. First, UAP56-marked cluster transcripts, their Rhino-marked genomic loci and Vasa-marked cytoplasmic processing centers congregate around the nuclear membrane, suggesting that transcript transfer in the germline happens in *cis*. This contrasts with the somatic event in which we have shown that Dot COM is distantly removed from the cluster loci. Second, UAP56 forms multiple nuclear foci in germline cells. Since the primary transcripts were not visualized *in situ*, it is unclear whether these foci correspond to multiple piRNA clusters. In the soma, our results suggest that all active clusters channel their transcripts to Dot COM. Thus it is an unlikely candidate for transporting transcripts to Dot COM.

Further characterization of Dot COM will require the identification of factors, *cis* and *trans* that are essential for Dot COM formation, and which will cement its essential function in piRNA-mediated transposon silencing. Our efforts have led to a simple RNA-FISH based assay for achieving this goal. For *cis* factors, genome manipulation could identify important elements required for targeting *COM* transcripts to Dot COM. Furthermore, recent RNAi screens identified a battery of new candidate genes involved in the piRNA pathway [21], [22]. Testing these genes will be employed for identifying factors essential for Dot COM formation. Finally, the availability of an ovarian somatic cell line [8] might enable unbiased biochemical purification of factors important for Dot COM function.

## Materials and Methods

### Drosophila stocks

The used fly strains were: *ISO1A*, *w^IR6^* and *Rev* lines, from the collection of the GReD; *KG00476*; *armi* [1]/*TM3* and *armi*[72.1]/*TM6*; *zuc* [HM27]/*CyO* and *zuc*[SG63]/*CyO*; RNAi lines were from Vienna Drosophila RNAi Centre: *armi* (103589KK), *zuc* (110430KK), *yb* (110056KK).

### Generation of mitotic clones in ovary

We used FRT/FLP techniques to generate clones of follicle cells homozygous for a null mutation in the *piwi* gene. Females with genotypes [*hs-FLP* 12; *FRT 42D piwi^2^*/*FRT 42D hs-myGFP*] and [*hs-FLP 70*; *ZAM-LTR*/+; *FRT 82B pntD88*/*FRT 82B Ubi-GFP*], respectively, were fed yeast for 24 hrs, heat shocked for 1 hr in a 37°C water bath and placed back in yeasted vials at 25°C for 60 hrs. Females [*hs-FLP 12*; *FRT 42D topCO*/*FRT 42D hs-myc*; *ZAM-LTR*/*+*] were then heat shocked again for 1 hr at 37°C to induce expression of the GFP tag. Females were dissected and fixed 1.5–2 hrs later.

### Construction of plasmids

To identify unique fragments present in the *COM* locus, the 180 kb sequence of this locus from the sequenced *ISO1A* strain were analyzed by CENSOR using Repbase [23]. Sequences homologous to TE were discarded. A BLAST was then performed with the remaining fragments to further detect any repetition within the *Drosophila* genome. Unique genomic fragments longer than 500 bp were used as probes. Although these genomic fragments may contain repeated regions <300 bp, the sequence of the full length probe is unique. These fragments were amplified from the *ISO1A* line and cloned into pGEMT easy vector. The primer pairs used are listed in Table S1.

### In Situ Hybridization

Riboprobes were synthesized by digestion of pGEMT easy plasmids with NcoI or SpeI enzyme, followed by *in vitro* transcription using Sp6 or T7 polymerase and digoxygenin or fluorescein labeled UTP (Roche), DNAse I treatment and purification.

DIG labeled *Het-A* probes are made with following primer sets with the PCR DIG probe synthesis kit from Roche: ACTACTGCAAGCACTTGTG and GTCTGCTCGTCGGATACTCA; AGCTCAGCAATCCTGAGCA and AGACGTTAGGGTTGAGTGTT; CAACAGACCACAGCCATCAT and TTTAACTTTGCTGGTGGAGGTAC.

Ovaries from 2-to 4-days-old flies were dissected in 0.2% Tween 20, phosphate buffered saline (PBT) fixed with 4% formaldehyde/PBT at RT for 30 min, rinsed three times with PBT and post-fixed 10 min in 4% formaldehyde/PBT. After washes in PBT and permeabilization (1 hr in PBS-0.3% Triton) prehybridization was done as follow: 10 min HYB- (Formamide 50%, SSC 5X, Tween 0.02%)/PBT 1:1, 10 min HYB-, 1 hr HYB+ (HYB- with yeast tRNA 0.5 μg/μl, heparin 0.25 mg/ml) at 60°C. Ovaries were hybridized overnight at 60°C with 1 μg riboprobe. Ovaries were then rinsed 20 min in HYB- and in HYB-/PBT 1:1 at 60°C then 4 times in PBT at RT before blocking 1 hr at RT in TNB 0.3% triton (Perkin-Elmer TSA kit) and immunodetection 1.30 hr at RT with anti-Dig-HRP (Roche) in TNB 0.2% tween. Ovaries were rinsed three times in PBT, incubated 10 min with TSA-Cy3 in amplification diluent (Perkin-Elmer), rinsed three times and stained with Hoescht. RNase A treatment was performed before the second step of fixation.

In double-RNA FISH experiments hybridization with both riboprobes was performed simultaneously. Staining of the first riboprobe was followed by a 10 min incubation with glycine 0.1 M–0.1% Tween HCL pH2.2 and washes in PBT followed by 1 hr incubation in PBT-0, 3% triton, washes and staining of the second riboprobe.

For DNA/RNA *in situ* hybridization, RNA staining was followed by treatment with 200 µg/ml RNase A for 2 hrs and ovaries were then transferred to FISH hybridization buffer containing 50% formamide, 4X SSC, 0,1% Tween 20, 0,1 M NaH2PO4. DNA was denatured 15 min at 80°C and hybridization was carried out O/N at 37°C. After washes, ovaries were first incubated with glycine 0.1 M–0.1% Tween HCL pH2.2 before washes and DNA staining. DNA probe, made of eleven PCR amplifications (Table S2) was labeled with digoxigenin using nick translation kit (Boehringer).

In RNA-immuno-FISH, RNA straining was followed by incubation with mouse anti-lamin antibody (ADL67-10, Hybridoma), mouse anti-Armitage antibody or rabbit anti-GFP antibody (sc-8334, Santa Cruz). Secondary antibodies coupled to Cy3 or Alexa-488 were used.

Three-dimensional images were acquired from stage 6 egg chambers on a Leica SP5 confocal microscope using a 40X objective.

## Supporting Information

Figure S1 **Scheme of** ***COM*** **piRNA cluster in** ***Drosophila*** **lines tested in this study (adapted from Malone & al (2009) Cell).** (**A**) The *COM* piRNA cluster is localized in the pericentromeric 20A region of the X-chromosome, upstream of the *DIP1* gene. It spans over 180 kb and harbours many defective transposons intermingled between each other mostly inserted in minus orientation (red dashes). Few are in a plus orientation (green dashes). It is proposed that piRNAs are processed from a single long precursor transcript produced by the locus. The 5′–3′ orientation of this transcript is indicated by an arrow above the locus. RNA probes used in this study are indicated by red rectangles above the *COM* transcript. Only antisense RNA probes are able to hybridize to this transcript. The DNA probe generated is shown as a blue rectangle underneath genomic DNA. (**B**) Scheme of *COM* genomic piRNA cluster in *W^IR6^* (top), *KG00476* (middle) and *Rev* (bottom) *Drosophila* lines, compared to the structure of the locus in *ISO1A* line (A). Yellow triangle indicates a *P*-element insertion. A dark bracket shows the genomic deletion affecting the *COM* locus in the *Rev* line.
(TIF)

Figure S2 **Riboprobes 527 and 654 are partially homologous to other piRNA clusters.** The scheme represents *Drosophila* chromosomes with pink and green arrows indicating genomic location of sequences recognized respectively by 527 and 654 riboprobes. Only sequences longer than 300 bp are shown. Gray rectangles and corresponding numbers underneath indicate some of the major piRNA clusters depicted in Malone *et al*. (2009). Black lines indicate minor piRNA clusters expressed in OSS cells and depicted in Lau *et al*. (2009). All these clusters produce piRNAs homologous to probes 527 or 654. A subset of these piRNAs match the genome at a unique position supporting their genomic origin.
(TIFF)

Figure S3 **HeT-A transcripts do not form foci in follicle cells.** A mid-stage ovariole from a *spnE* mutant ovary is shown. HeT-A RNA FISH detected abundant transcripts (signals in red) accumulating in the oocyte. Yet, HeT-A transcripts did not form foci in follicle cells. DNA signals are in blue. In wild type ovaries, HeT-A transcripts displayed no focus in follicle cells either (not shown).
(PDF)

Figure S4 **Dot COM does not co-localize with nucleolus, cajal bodies or histones bodies.** Immuno-RNA FISH staining in ovarian somatic follicle cells of *ISO1A* line with antisense 508 riboprobe and antibodies against fibrillarin (A), coilin (B) and Lsm11 (C) proteins (anti-coilin and anti-Lsm11 antibodies were kindly provided by J. Gall) that mark respectively the nucleolus, cajal bodies and histone core bodies. Anti-lamin antibody marks the nuclear membrane. DNA is stained in blue.
(TIFF)

Figure S5 **Yb-bodies are disrupted in ovarian follicle cells expressing** ***Yb*** **RNAi.** Immuno-RNA FISH staining with antisense 508 riboprobe (red) and anti-armi antibody (green) in ovarian somatic follicle cells of *ISO1A* line and RNAi mutant for *Yb*. DNA is stained in blue. In RNAi *Yb* mutants, Armi does not accumulate in 1 or 2 cytoplasmic foci but is dispersed within the cytoplasm whereas COM 508 transcript still accumulates in a single nuclear dot.
(TIFF)

Table S1 **List of primers used to PCR amplify genomic fragments used as RNA probes.** Positions of amplified DNA were determined by mapping genomic positions to the Release 4.45 assembly. Genomic fragments used for riboprobes were amplified from the *ISO1A* line and cloned into pGEMT easy vector. Primers are indicated in 5′ to 3′ orientation.
(TIF)

Table S2 **List of primers used to PCR amplify genomic fragments used as DNA probe.** Positions of amplified DNA were determined by mapping genomic positions to the Release 4.45 assembly. Primers are indicated in 5′ to 3′ orientation.
(TIFF)

Video S1 **Transcripts from** ***COM*** **locus localize in the nucleus, close to the nuclear membrane.** Movie of 3D imaging of ovarian follicle cells stained with *COM* riboprobe 508 (red) and anti-lamin antibody (green). DNA is indicated in blue.
(MOV)
