# Supplementary figures and images for: “Dot COM”, a Nuclear Transit Center for the Primary piRNA Pathway in Drosophila
Source: PLoS One. 2013 Sep 9;8(9):e72752. doi: 10.1371/journal.pone.0072752 (PMC3767702; doi:10.1371/journal.pone.0072752)

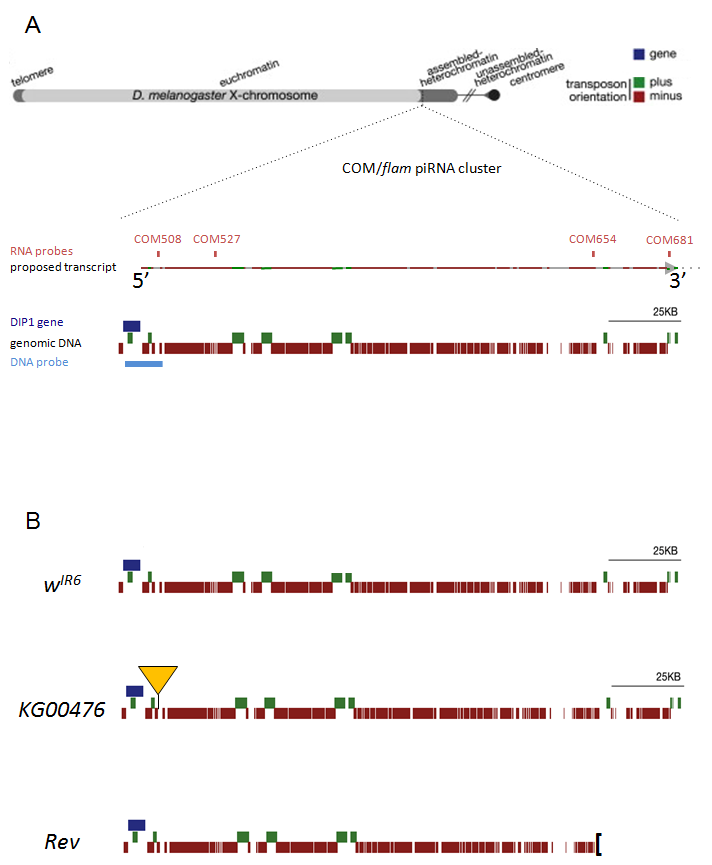

Supplement: Figure S1 — Scheme of COM piRNA cluster in Drosophila lines tested in this study (adapted from Malone & al (2009) Cell). (A) The COM piRNA cluster is localized in the pericentromeric 20A region of the X-chromosome, upstream of the DIP1 gene. It spans over 180 kb and harbours many defective transposons intermingled between each other mostly inserted in minus orientation (red dashes). Few are in a plus orientation (green dashes). It is proposed that piRNAs are processed from a single long precursor transcript produced by the locus. The 5′–3′ orientation of this transcript is indicated by an arrow above the locus. RNA probes used in this study are indicated by red rectangles above the COM transcript. Only antisense RNA probes are able to hybridize to this transcript. The DNA probe generated is shown as a blue rectangle underneath genomic DNA. (B) Scheme of COM genomic piRNA cluster in WIR6 (top), KG00476 (middle) and Rev (bottom) Drosophila lines, compared to the structure of the locus in ISO1A line (A). Yellow triangle indicates a P-element insertion. A dark bracket shows the genomic deletion affecting the COM locus in the Rev line. (TIF) [file pone.0072752.s001.tif]

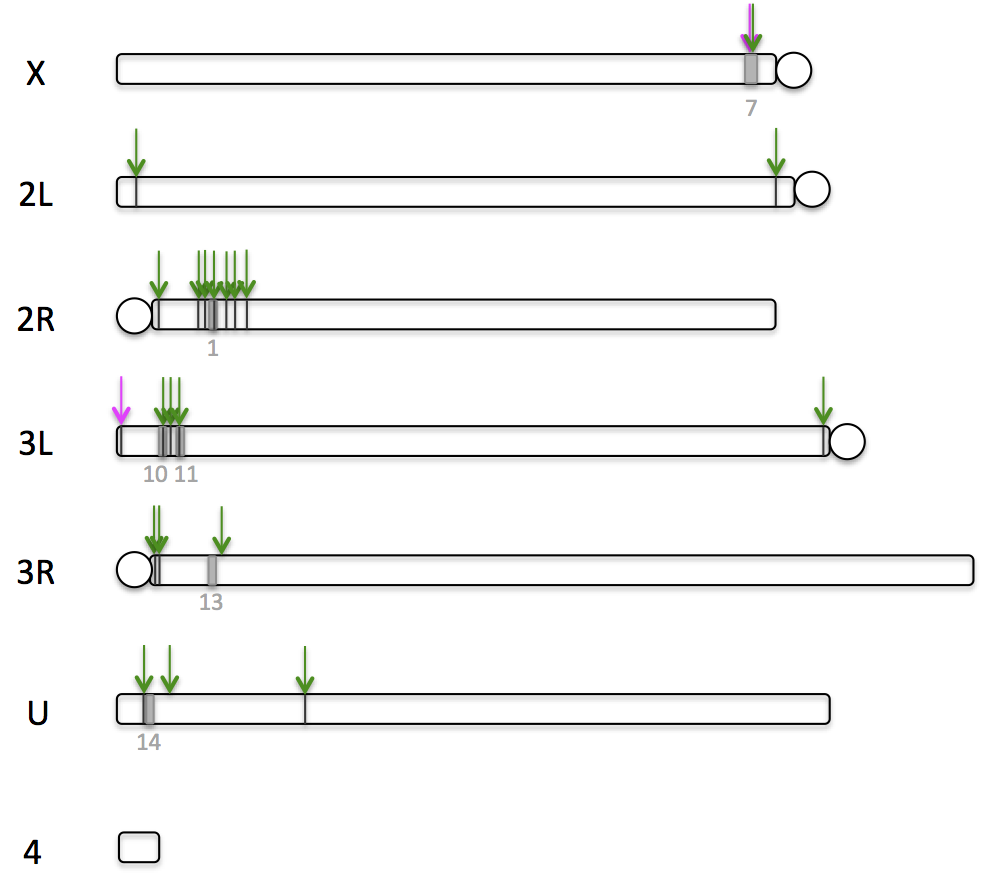

Supplement: Figure S2 — Riboprobes 527 and 654 are partially homologous to other piRNA clusters. The scheme represents Drosophila chromosomes with pink and green arrows indicating genomic location of sequences recognized respectively by 527 and 654 riboprobes. Only sequences longer than 300 bp are shown. Gray rectangles and corresponding numbers underneath indicate some of the major piRNA clusters depicted in Malone et al. (2009). Black lines indicate minor piRNA clusters expressed in OSS cells and depicted in Lau et al. (2009). All these clusters produce piRNAs homologous to probes 527 or 654. A subset of these piRNAs match the genome at a unique position supporting their genomic origin. (TIFF) [file pone.0072752.s002.tif]

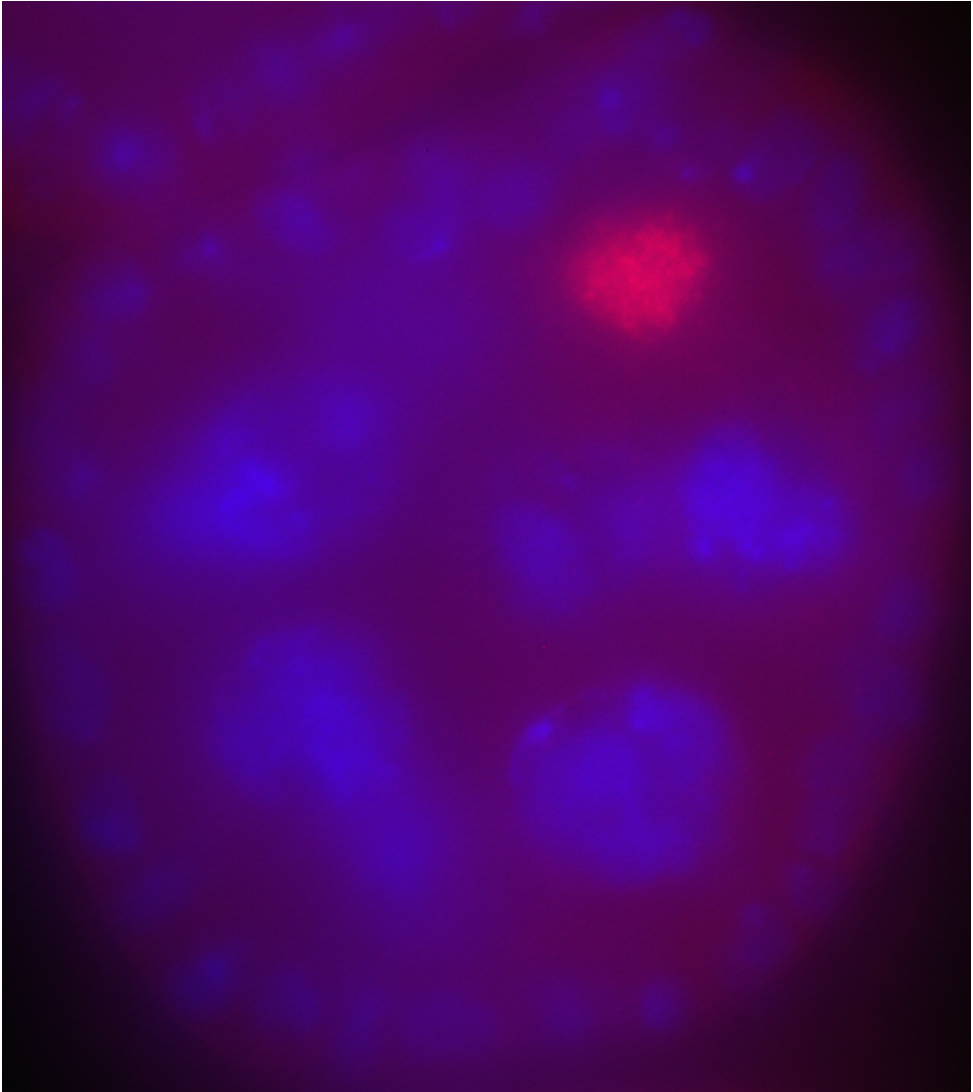

Supplement: Figure S3 — HeT-A transcripts do not form foci in follicle cells. A mid-stage ovariole from a spnE mutant ovary is shown. HeT-A RNA FISH detected abundant transcripts (signals in red) accumulating in the oocyte. Yet, HeT-A transcripts did not form foci in follicle cells. DNA signals are in blue. In wild type ovaries, HeT-A transcripts displayed no focus in follicle cells either (not shown). (PDF) [file pone.0072752.s003.pdf]

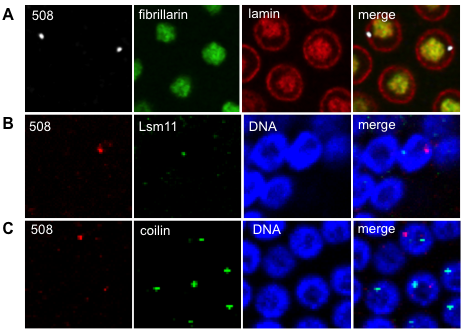

Supplement: Figure S4 — Dot COM does not co-localize with nucleolus, cajal bodies or histones bodies. Immuno-RNA FISH staining in ovarian somatic follicle cells of ISO1A line with antisense 508 riboprobe and antibodies against fibrillarin (A), coilin (B) and Lsm11 (C) proteins (anti-coilin and anti-Lsm11 antibodies were kindly provided by J. Gall) that mark respectively the nucleolus, cajal bodies and histone core bodies. Anti-lamin antibody marks the nuclear membrane. DNA is stained in blue. (TIFF) [file pone.0072752.s004.tif]

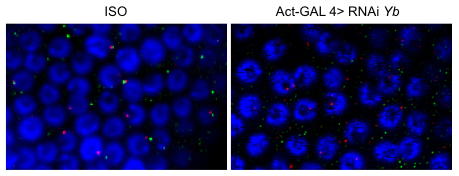

Supplement: Figure S5 — Yb-bodies are disrupted in ovarian follicle cells expressing Yb RNAi. Immuno-RNA FISH staining with antisense 508 riboprobe (red) and anti-armi antibody (green) in ovarian somatic follicle cells of ISO1A line and RNAi mutant for Yb. DNA is stained in blue. In RNAi Yb mutants, Armi does not accumulate in 1 or 2 cytoplasmic foci but is dispersed within the cytoplasm whereas COM 508 transcript still accumulates in a single nuclear dot. (TIFF) [file pone.0072752.s005.tif]

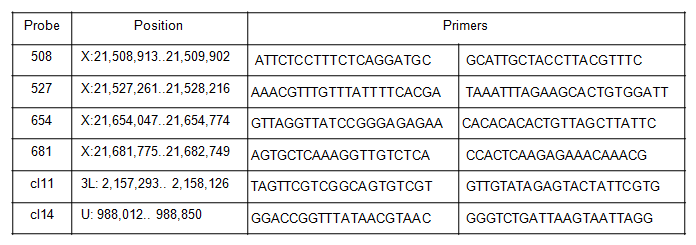

Supplement: Table S1 — List of primers used to PCR amplify genomic fragments used as RNA probes. Positions of amplified DNA were determined by mapping genomic positions to the Release 4.45 assembly. Genomic fragments used for riboprobes were amplified from the ISO1A line and cloned into pGEMT easy vector. Primers are indicated in 5′ to 3′ orientation. (TIF) [file pone.0072752.s006.tif]
